# Characterization of the p.L145F and p.S135N Mutations in SOD1: Impact on the Metabolism of Fibroblasts Derived from Amyotrophic Lateral Sclerosis Patients

**DOI:** 10.3390/antiox11050815

**Published:** 2022-04-22

**Authors:** Elisa Perciballi, Federica Bovio, Jessica Rosati, Federica Arrigoni, Angela D’Anzi, Serena Lattante, Maurizio Gelati, Fabiola De Marchi, Ivan Lombardi, Giorgia Ruotolo, Matilde Forcella, Letizia Mazzini, Sandra D’Alfonso, Lucia Corrado, Mario Sabatelli, Amelia Conte, Luca De Gioia, Sabata Martino, Angelo Luigi Vescovi, Paola Fusi, Daniela Ferrari

**Affiliations:** 1 Department of Biotechnology and Biosciences, University of Milano-Bicocca, P.zza della Scienza, 2, 20126 Milan, Italy; e.perciballi@campus.unimib.it (E.P.); federica.bovio@unimib.it (F.B.); federica.arrigoni@unimib.it (F.A.); ivan.lombardi@unimib.it (I.L.); matilde.forcella@unimib.it (M.F.); luca.degioia@unimib.it (L.D.G.); angelo.vescovi@unimib.it (A.L.V.); 2 Cellular Reprogramming Unit, Fondazione IRCCS Casa Sollievo della Sofferenza, Viale dei Cappuccini 1, 71013 San Giovanni Rotondo, Italy; j.rosati@css-mendel.it (J.R.); a.danzi@css-mendel.it (A.D.); r.giorgia1997@libero.it (G.R.); 3 Section of Genomic Medicine, Department of Life Sciences and Public Health, Università Cattolica del Sacro Cuore, Largo Francesco Vito, 1, 00168 Rome, Italy; serena.lattante@unicatt.it; 4 Unit of Medical Genetics, Department of Laboratory and Infectious Disease Sciences, Fondazione Policlinico Universitario A. Gemelli IRCCS, Largo A. Gemelli 8, 00168 Rome, Italy; 5 UPTA Unit, Fondazione IRCCS Casa Sollievo della Sofferenza, Viale dei Cappuccini 1, 71013 San Giovanni Rotondo, Italy; m.gelati@css-mendel.it; 6 ALS Centre Maggiore della Carità Hospital and Università del Piemonte Orientale, 28100 Novara, Italy; fabiolademarchi@gmail.com (F.D.M.); letizia.mazzini@uniupo.it (L.M.); 7 Department of Health Sciences, Center on Autoimmune and Allergic Diseases (CAAD), UPO, University of Eastern Piedmont, 28100 Novara, Italy; sandra.dalfonso@med.uniupo.it (S.D.); lucia.corrado@med.uniupo.it (L.C.); 8 Adult NEMO Clinical Center, Unit of Neurology, Department of Aging, Neurological, Orthopedic and Head-Neck Sciences, Fondazione Policlinico Universitario A. Gemelli IRCCS, Largo A. Gemelli 8, 00168 Rome, Italy; mario.sabatelli@unicatt.it (M.S.); amelia.conte@centrocliniconemo.it (A.C.); 9 Section of Neurology, Department of Neuroscience, Faculty of Medicine and Surgery, Università Cattolica del Sacro Cuore, Largo Francesco Vito, 1, 00168 Rome, Italy; 10 Department of Chemistry, Biology and Biotechnology, University of Perugia, Via del Giochetto, 06123 Perugia, Italy; sabata.martino@unipg.it; 11 Fondazione IRCCS Casa Sollievo della Sofferenza, Viale dei Cappuccini 1, 71013 San Giovanni Rotondo, Italy

**Keywords:** amyotrophic lateral sclerosis, ALS, p.L144F, p.S134N, SOD1 mutations, seahorse, energetic metabolism, p.L145F, p.S135N, mitochondria

## Abstract

Amyotrophic lateral sclerosis (ALS) is a fatal neurodegenerative disease characterized by the loss of the upper and lower motor neurons (MNs). About 10% of patients have a family history (familial, fALS); however, most patients seem to develop the sporadic form of the disease (sALS). *SOD1* (Cu/Zn superoxide dismutase-1) is the first studied gene among the ones related to ALS. Mutant SOD1 can adopt multiple misfolded conformation, lose the correct coordination of metal binding, decrease structural stability, and form aggregates. For all these reasons, it is complicated to characterize the conformational alterations of the ALS-associated mutant SOD1, and how they relate to toxicity. In this work, we performed a multilayered study on fibroblasts derived from two ALS patients, namely SOD1^L145F^ and SOD1^S135N^, carrying the p.L145F and the p.S135N missense variants, respectively. The patients showed diverse symptoms and disease progression in accordance with our bioinformatic analysis, which predicted the different effects of the two mutations in terms of protein structure. Interestingly, both mutations had an effect on the fibroblast energy metabolisms. However, while the SOD1^L145F^ fibroblasts still relied more on oxidative phosphorylation, the SOD1^S135N^ fibroblasts showed a metabolic shift toward glycolysis. Our study suggests that SOD1 mutations might lead to alterations in the energy metabolism.

## 1. Introduction

Amyotrophic lateral sclerosis (ALS) is a progressive neurodegenerative disorder, characterized by the loss of the upper motor neurons (MNs) in the motor cortex, as well as lower MNs at the bulbar and spinal level [1]. ALS determines skeletal muscle paralysis and leads to patient death, usually in 3–5 years, and mostly related to respiratory failure [2]. At least 25% of ALS patients carry a genetic mutation which is likely to be the cause of the disease [3,4,5]. Recent advances in the field and improved technologies for gene mapping and DNA analysis have facilitated the identification of several “ALS genes” and more than 150 genetic variants associated with increased risk for the disease [6]. The *SOD1* gene encodes for copper/zinc ion-binding SOD, and it is the first mutated gene that has been associated with ALS [7]. Mutations in the SOD1 gene account for approximately 15–30% of cases of fALS and 2% of sALS [8].

Wild-type SOD1 is usually stable and mostly localized in the cytoplasm: however, a small fraction has been found in the intermembrane space of mitochondria [9]. Finally, it has been shown that, in response to an increase in reactive oxygen species (ROS) levels, wild-type SOD1 can also be located in the nucleus, where it might regulate the expression of oxidative resistance and repair genes [10,11]. Mutant SOD1 can adopt multiple misfolded conformations and might interfere with cell functionality through several mechanisms. It has been shown that misfolded SOD1 can accumulate on the cytoplasmic faces of mitochondria, leading to mitochondrial dysfunction and cell death, as well as within the ER in the spinal cord of transgenic mice, where it forms insoluble high molecular weight aggregates [12,13]. Mutant SOD1-induced toxicity might also have an effect on the microglial and glial cells; in fact, the accumulation of mutated SOD1 protein in these cell types could induce the expression of pro-inflammatory cytokines [14]. These studies, taken together, provide a complex and dynamic picture of SOD1 functionality and suggest that the physiological role of SOD1, and the toxicity of its mutated forms, goes beyond its enzymatic role and its cytoplasmic localization. The mechanisms through which mutated SOD1 can contribute to ALS progression still need to be fully elucidated.

Throughout the *SOD1* gene, over 185 disease-associated allelic variants have been identified [15]. The missense point variant p.S135N in the exon 5 of the *SOD1* gene [16] is associated with a rapid progressive loss of motor function with predominant lower motor neuron manifestations [17]. This mutation causes substitutions in the electrostatic loop that, in turn, lead to impaired metal binding with disordered electrostatic and zinc-binding loops [13,18].

The SOD1 p.L145F mutation is located in the fifth exon of the gene [19]: it is typical of the Balkan regions and shows peculiar clinical features [20]. The impact this mutation has on the SOD1-protein is still poorly described; however, Masè et al. [21] have reported a decrease in SOD1 dismutase activity.

An increasing number of studies are investigating alterations in the energy metabolism of ALS patients and study models in order to better understand their role in disease progression. MNs are extremely vulnerable to ATP depletion. As such, energetic imbalance might have a severe effect on this type of cell. In addition, the degeneration of mitochondria, which is the main site of ROS production in the cell, has been linked to cell death mechanisms in MNs [22]. Deficiencies in the activity of the different complexes of the electron transport chain (ETC) have been described in the spinal cord and muscle of both fALS and sALS patients, and in cell and animal models of the disease [23]. Recent studies suggest that the toxicity of the fALS mutant SOD1 might be caused by its selective recruitment to the mitochondria. Furthermore, the presence of mutSOD1s leads to the impairment of the respiratory chain and a shift in the mitochondrial redox balance towards a more oxidizing state measured as the ratio between reduced glutathione and its oxidized form (GSH/GSSG ratio) [18]. Glutathione depletion can enhance oxidative stress; indeed, as GSH synthesis requires ATP, the deficiency of energy supplied by mitochondria is likely to affect the cellular turnover of GSH [24].

In the present study, we investigated the bioenergetic profile of fibroblasts derived from two patients (namely SOD^L145F^ and SOD^S135N^) bearing two non-classical mutations of SOD1 (p.L145F and p.S135N, respectively). The bioinformatic analysis predicted different effects of the mutations on protein stability and functionality: p.L145F was predicted to lightly affect the enzymatic core of the protein, most likely impairing SOD1 ability to form dimers, while the p.S135N was considered to have a more destabilizing effect on the metal-binding core, also reported in previous studies. Of note, both mutations could cause an imbalance in the energy metabolism of the cells with an altered mitochondrial functionality and, for the p.S135N, a shift toward glycolysis.

Our study suggests that the two mutations of SOD1 described, namely, p.S135N and p.L145F, may have an effect on cell energy metabolism. Our work describes an experimental setting to study patients’ fibroblasts in combination with bioinformatics and Seahorse analyses. Our data might be helpful for the characterization of the ALS cellular phenotype: the technology described provides the tools to further investigate such mechanisms in MNs and glial cells derived from patients [25].

## 2. Materials and Methods

### 2.1. Molecular Modeling

The change in protein stability (ΔΔG, kcal/mol) of site-specific mutations of a high-resolution (1.07 Å) human Cu-Zn SOD1 homodimer structure (PDB ID: 2C9V) was predicted using the PoPMuSiC protein stability calculator [26], which has emerged from previous benchmarks as the best method to predict the SOD1 stability changes of mutations [27]. To further corroborate the ΔΔG value prediction, we repeated the experiments with four other different (in terms of algorithms/scoring functions used) recent web-server-based tools, namely, SDM [28], MAESTROweb [29], PremPS [30] and INPS-3D [31]. All the above-mentioned calculations were performed considering a single monomer of SOD1. For the structural inspection of wild-type vs. mutated forms, mutant 3D models were built starting from the 2C9V structure. Firstly, the latter was processed with Schrodinger’s Protein Preparation Wizard [32], as implemented in the Maestro 2021-4 interface [33], which adjusts the raw experimentally derived structure. It was used to assign missing H atoms, disulphide bonds, optimal formal oxidation and protonation states (at pH 7) to optimize H-bonds and to run a restrained geometry optimization. The latter allows H atom positions to be fully optimized and heavy atoms to be sufficiently moved (0.3 Å RMSD) to relax strained degrees of freedom and steric clashes. In silico mutagenesis was then induced using the BioLuminate modeling platform [34]. The obtained models were refined with both side-chain predictors and local minimization tools (with a cutoff of 5 Å around the mutated site). Hydrogen bonds patterns and possible steric clashes of mutated side-chains with proximal residues were analyzed with both Schrodinger Maestro software and the FindHBond and FindClash functions of UCSF Chimera [35]. Images were generated using PyMOL [36].

### 2.2. Collection of Skin Biopsies

Dermal skin biopsies from one healthy volunteer (CTRL) and ALS patients with pathogenic variants of SOD1 (SOD1^S135N^ and SOD1^L145F^) were collected using a disposable biopsy punch of 4 or 5 mm in diameter, placed in a sterile solution. Skin fragments were then transferred to a sterile hood and hand cut in smaller fragments and plated on a 35 mm tissue-culture dish with fetal bovine serum (FBS—Sigma Aldrich, St. Louis, MO, USA) at 37 °C, 5% CO_2_, overnight. The day after, dermal fragments were transferred to Dulbecco’s Modified Eagle Medium (DMEM) high glucose (Sigma Aldrich, St. Louis, MO, USA) supplemented with 20% FBS (Sigma Aldrich, St. Louis, MO, USA), 1% L-glutamine (Sigma-Aldrich, St. Louis, MO, USA), penicillin-streptomycin (Sigma Aldrich, St. Louis, MO, USA) and non-essential amino acids (Sigma Aldrich, St. Louis, MO, USA) and cultured until fibroblasts, sprouting out from the skin fragments, reached confluency. Then, cells were treated as a normal primary fibroblast culture.

### 2.3. Primary Skin Fibroblast Cultures and Growth Curves

All fibroblasts were cultured in DMEM high glucose supplemented with 20% FBS, 1% L-glutamine, penicillin-streptomycin (Sigma Aldrich, St. Louis, MO, USA) and non-essential amino acids (Sigma Aldrich, St. Louis, MO, USA). Cellular growth was monitored as follows: 30,000 cells were plated in 12-well (3.8 cm^2^) multi-well plates and counted after 24, 48, 72, and 96 h in culture. All experiments were performed at least in triplicate.

### 2.4. SOD1 Immunofluorescence Staining

Cells were seeded on glass coverslips at the optimal density of 1 × 10^3^ and were fixed with 4% paraformaldehyde. Unspecific binding sites on fixed cells were blocked with 10% Bovine Serum Albumin (BSA—Sigma Aldrich, St. Louis, MO, USA), 1% normal goat serum (NGS—Sigma Aldrich, St. Louis, MO, USA) and permeabilized with 0.1% Triton X-100 for 1 h at RT in 1X phosphate-buffered saline with Ca^2+^ and Mg^2+^ (PBS++—Sigma Aldrich, St. Louis, MO, USA), and incubated overnight at 4 °C with the primary antibody: anti-SOD1 (1:400, Santa Cruz Biotechnology, CA, USA). Finally, after incubation with Alexa-Fluor-594 nm, secondary antibodies were conjugated (InvitrogenTM, Grand Island, NY, USA) for 1 h at RT, coverslips with cells were mounted, and nuclei were counterstained with DAPI. The immunofluorescence images Figure 2 represent the “Maximum Projection” of a z-stack composed of 7/12 steps (1 µm each) for images taken at lower magnification and 10 steps (0.3 µm each) for images taken at higher magnification, and were acquired with a Nikon A1R confocal microscope of the PMib centre at the University of Milano-Bicocca (Nikon, Tokyo, Japan). Levels were equally optimized for the control and patient fibroblasts.

### 2.5. Detection of Intracellular Cytosolic Reactive Oxygen Species (ROS)

Intracellular reactive oxygen species (ROS) were detected via the oxidation of 2′,7′-dichlorofluorescin diacetate (H2DCFDA) (Sigma Chemical Co., St. Louis, MO, USA). H 2 DCF-DA is a diacetate and nonfluorescent form of DCF, which is cell permeable; after entering the cytoplasm, intracellular esterases cleave the two ester bonds of H 2 DCF-DA in an ROS-dependent reaction, and the fluorescent molecule DCF is released [37]. Fibroblasts were seeded in 96-well black microtiter plates at a density of 1 × 10^4^ cells/well, cultured in complete medium, and incubated after 24 h with 5 μM H2DCFDA in PBS for 30 min in the dark at 37 °C. Plates were rinsed in PBS twice, and cells were treated with 1 mM H_2_O_2_ for 30 min as a positive control. Fluorescence (λem = 485 nm/λex = 535 nm) was measured at 37 °C using a fluorescence microtiter plate reader (VICTOR X3, PerkinElmer, Akron, OH, USA).

### 2.6. Glutathione Detection

Fibroblasts were seeded in 96-well microtiter plates at a density of 1 × 10^4^ cells/well for total glutathione (GSH) content measurement, as described in Bovio et al., 2021 [38]. For total GSH content, 50 µL of each cell lysate was used for all of the cell lines. All chemicals were supplied by Merck KGaA, Darmstadt, Germany.

### 2.7. Oxygen Consumption Rate and Extracellular Acidification Rate Measurements

Oxygen consumption rate (OCR) and extracellular acidification rate (ECAR) were assessed using the Agilent Seahorse XFe96 Analyzer with fibroblasts from the healthy volunteer and the patients carrying the mutations.

The cells were seeded in Agilent Seahorse 96-well XF cell culture microplates at a density of 4 × 10^4^ cells per well in 180 μL of growth medium, and were allowed to adhere for 24 h in a 37 °C humidified incubator with 5% CO_2_. Before running the assay, the Seahorse XF Sensor Cartridge was hydrated and calibrated with 200 μL of Seahorse XF Calibrant Solution in a non-CO_2_ 37 °C incubator to remove CO_2_ from the media that would otherwise interfere with the pH-sensitive measurements. Subsequently, the Agilent Seahorse XF Cell Energy Phenotype Test Kit, Mito Stress Test Kit, ATP Rate Assay Kit and Glycolytic Rate Assay Kit and Glycolysis Stress Test were performed according to manufacturer protocols.

All kits and reagents were purchased from Agilent Technologies (Santa Clara, CA, USA).

### 2.8. Statistical Analysis

Three biological replicates for each experiment were performed, and each single biological replicate was carried out in four technical replicates. The one-way and two-way ANOVA tests with Tukey’s correction were used to evaluate statistical significance within the different samples. Results were considered statistically significant at *p* < 0.05 (*), *p* < 0.01 (**) and *p* < 0.001 (***).

## 3. Results

### 3.1. Clinical Evaluation of the Patients

#### 3.1.1. SOD1^L145F^ Patient

The p.L145F mutation (also known as p.L144F) was carried by a female patient recruited from the Tertiary ALS Center at the Department of Neurology, University of Piemonte Orientale, Novara, Italy.

This patient received ALS diagnosis according to the El Escorial criteria [39]. She suffers from a familial form of ALS, with slow disease progression. Her remote history was unremarkable. She reported two relatives affected by motor neuron disease (a paternal grandmother and a younger brother).

She first developed symptoms at the age of 47: she experienced weakness and muscle atrophy in the right foot, which subsequently diffused proximally and moved to the left leg within a few months. During her first examination, twelve months after the onset of the first symptoms, her ALS Functional Rating Scale—Revised (ALSFRS-R) score was 40/48 (12-10-6-12). She started treatment with riluzole (100 mg/day). The worsening of her functional status progressed slowly, mainly with predominant lower motor neuron signs. After four years, she started non-invasive ventilation for dyspnea. Five years after from symptom onset she is not ambulatory, with mild dysarthria and dysphagia.

Notably, three years from diagnosis, the patient developed severe urinary and fecal incontinence. No cognitive impairment was developed over the progression of the disease.

#### 3.1.2. SOD1^S135N^ Patient

The p.S135N missense variant (also known as p.S134N) was identified in a male ALS patient, who was evaluated in a referral ALS Center at the Department of Neurology, Catholic University of the Sacred Heart in Rome.

The patient had a familial history of ALS: his maternal uncle died of ALS after two years of the disease; no other family members were affected by ALS.

At the age of 52, he noted progressive weakness of the proximal muscles of his lower limbs. At the first neurological examination, 6 months after disease onset, he had atrophy and weakness of the proximal muscles of the lower limbs and fasciculations in all limbs; no upper limbs weakness, respiratory involvement, or bulbar signs were present. During the course of the disease, lower motoneuron signs were predominant. About 18 months after the first symptoms were noted, he started non-invasive mechanical ventilation (NIV) and cough-assisted machine therapy. Two years after disease onset, he developed severe tetraparesis, and six months later mild dysarthria. His cognitive functions were normal. He died 34 months after the onset of the disease, of respiratory failure.

### 3.2. Bioinformatic Analysis: p.L145F and p.S135N Differently Affect SOD1 Structural Properties

**Figure 1:**
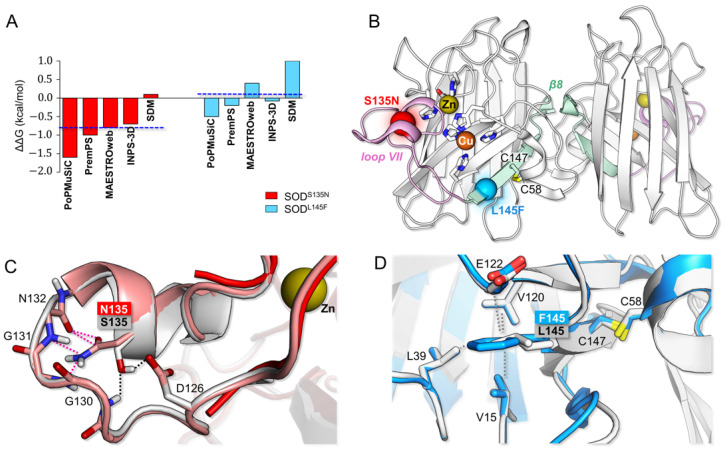
Structural properties of p.S135N and p.L145F mutants. (**A**) Theoretical protein stability changes upon mutation (expressed as folding free energy variation ΔΔG = ΔG (wild type) − ΔG (mutant), in kcal/mol). For each mutation, the average ΔΔG resulting from the five tested methods is indicated by a horizontal blue–dashed line. (**B**) Cartoon representation of SOD1 homodimeric structure (2C9V). S135N and L145F positions are indicated by a red and a light blue sphere, respectively (in the single monomeric unit). Loop VII (electrostatic loop) is highlighted in pink, while the β8 sheet is in pale green. Side-chains of selected residues have been represented as sticks: C58, C147 and Cu and Zn first coordination sphere. (**C**) Superimposition of wild-type crystal structure (2C9V, in grey), p.S135N in silico model (in salmon), and crystal structure (1OZU, in red), with a focus on the electrostatic loop portion 123–142. In 1OZU, part of the latter is absent. H-bonds of S135 with D126 and G130 are reported as black–dotted lines. Steric clashes with residues 130–132, originating from S135 substitution by N in the in silico model, are reported as pink–dotted lines. (**D**) Superimposition of wild-type crystal structure (2C9V, in grey) and p.L145F in silico model (in light blue), with focus on site 145 in β8. Hydrophobic contacts of both L (in the wild type) and F (in the mutant) are indicated as grey–dotted lines. The proximal disulphide bond has been used as evidence. Atom coloring: O = red; N = blue; S = yellow; H = white; C = grey, pale green, salmon or light blue, according to the color of the corresponding secondary structure element.

The analysis of the impact of specific mutations on the stability of SOD1 is a first step in order to be able to link SOD1 mutant structures to phenotypes [40]. In this context, the theoretical evaluation of folding free energy of the wild type, and its change (ΔΔG) when a point mutation is present, has been found to be an excellent strategy to predict whether a mutation could be favorable in terms of protein stability [27]. ΔΔG values for the p.L145F and p.S135N mutations, calculated with PoPMuSiC and other four different algorithms, are presented in Figure 1A. We predicted that p.S135N would result in greater destabilization (ΔΔG = −1.6–0.2 kcal/mol, avg ΔΔG = −0.8 kcal/mol) as compared to p.L145F (ΔΔG = −0.4–1.0 kcal/mol, avg ΔΔG = 0.1 kcal/mol). The different results obtained can be justified by the localization of the mutated residues, as detected on the enzyme 3D structure. Our findings are in accordance with previously published work, as detailed later.

S135 is located rather in proximity to the Cu-Zn site, being in the middle of a small helix (residues 133–138) that is found in the loop VII, referred to as the “electrostatic loop” (residues 123–145) (Figure 1B). The electrostatic loop is essential for protein function and enables the positioning of substrates in a productive orientation toward the metal centers [41]. The in silico model of p.S135N clearly shows that N causes severe steric clashes with loop residues 130–132 (Figure 1C). Additionally, two H-bonds formed by S135 with D126 and G130 in the wild type are disrupted upon substitution with N. Therefore, p.S135N could lead to a conformational change in the electrostatic loop, destabilizing the canonical helical folding. This is confirmed by the crystal structure of the p.S135N-mutated protein (PDB ID: 1OZU) in which the 133–135 portion of the loop is not visible, suggesting its increased disorder upon mutation. The significant impact of p.S135N on SOD1 stability also leads to noticeable alterations of its function. Indeed, p.S135N mutation has been previously associated with defective metal-binding and a 3–5-fold lower catalytic activity with respect to the wild type [42]. Furthermore, increased flexibility and non-native conformation of the electrostatic loop have been related to an unusual gain-of-interaction by previous experimental [43,44] and recent theoretical [45,46] investigations, leading to abnormal aggregation.

L145 is about 11 Å far from Cu in the Cu-Zn center and it is located at the edge of the β8 strand (at the conjunction with the electrostatic loop), which is involved in the dimer interface packing (Figure 1B). ΔΔG calculations suggest that L145F should be tolerated and should not be destabilizing (Figure 1A). In fact, in the 3D model of the mutated protein, the F side-chain in the mutant is involved in the same Van der Waals interactions of L in the wild type, with V15, L39, V120 and E122 (Figure 1D). This is in line with previous results [47,48], showing that L145F has no effect on monomer stability. The same investigation indicated instead that it is detrimental for homodimer formation. p.L145F thus has a long-range effect at the dimer interface, which is difficult to infer from the structure of the 3D model. It is possible that p.L145F, which increases the side-chain volume to be accommodated within the β barrel hydrophobic core, may alter the dynamic behavior of the protein, affecting the vicinal C147-C58 disulphide bond, which is crucial for SOD1 correct dimerization [49,50].

### 3.3. Analysis of ALS Patient-Derived Fibroblasts: SOD1^L145F^ and SOD1^S135N^ Cells

#### 3.3.1. Morphology of ALS-Fibroblasts

**Figure 2:**
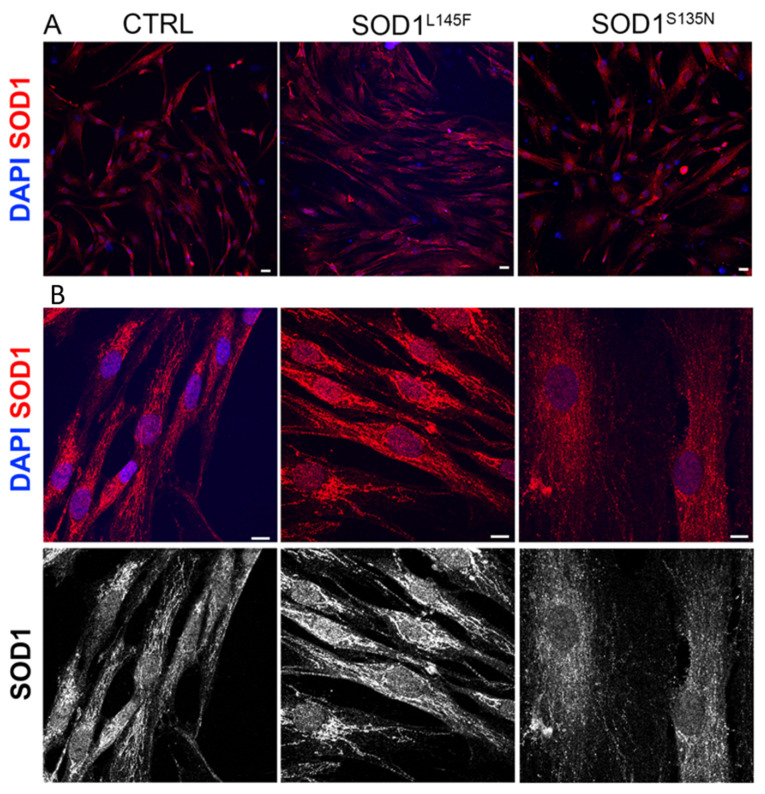
Representative immunofluorescence of SOD1 expression (anti-SOD1; red); nuclei are stained in blue (4′,6-diamidino-2-phenylindole, DAPI). Scale bars: (**A**) = 25 µm; (**B**) = 10µm.

The fibroblasts derived from the patients differed from the healthy ones from a morphological point of view (Figure 2A,B). While the control cells had a typical fusiform and elongated shape, the ones derived from the ALS patients showed a flat shape, and were bigger in size (especially SOD1^S135N^ ones).

The immunofluorescence analysis of SOD1 revealed widespread distribution in the control and mutated fibroblasts (Figure 2A,B). However, in some ALS patients’ fibroblasts we observed that SOD1 localized more in the perinuclear cytoplasm compared to what was observed in fibroblasts from the healthy donor, albeit not in all cells (Figure 2B).

#### 3.3.2. ALS Fibroblasts Showed Increased Proliferation Rate Compared to the Control

**Figure 3:**
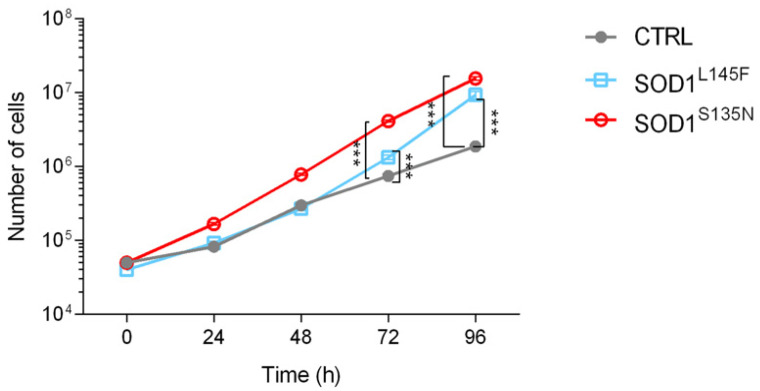
Growth curves of SOD1 mutants and control fibroblasts. Each point represents the mean of at least three independent experiments and results are shown as the mean ± SEM. Statistical significance: *** *p* < 0.001.

To assess the impact the mutations had on the functional properties of the fibroblasts, we first compared the proliferative ability of SOD1^S135N^, SOD1^L145F^, and the healthy fibroblasts. Growth curve analysis (Figure 3) showed that both the SOD1 mutations were associated with increased proliferation rates compared to the control fibroblasts. Statistically significant differences were observed at the two timepoints considered (72 and 96 h).

#### 3.3.3. ALS Fibroblasts Show Reduced Anti-Oxidant Potential

Given the fact that oxidative stress is most likely involved in ALS etiology [51] and that it might also drive cell cycle activation through the ERK1/2 pathway [52], we analyzed the total ROS levels.

**Figure 4:**
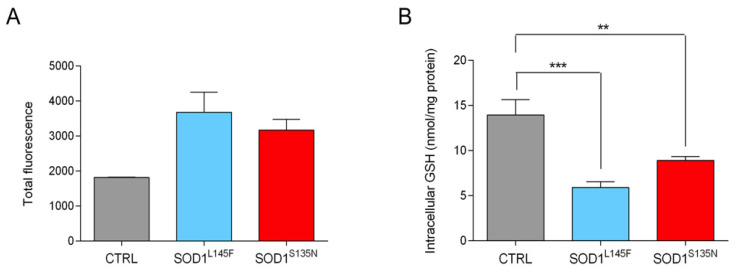
Analysis of oxidative stress. Detection of the total intracellular ROS levels (**A**) and total intracellular glutathione levels (**B**). Data are shown as the mean ± SEM. Statistical significance: ** *p* < 0.01 and *** *p* < 0.001.

The measurements of DCF fluorescence (Figure 4A) showed higher levels of ROS in both cell lines carrying the SOD1 mutations compared to the control fibroblasts; however, the differences observed were not statistically significant.

To perform a more comprehensive assessment of whether the mechanisms regulating the homeostasis between ROS and antioxidant defenses were affected [53], we measured the levels of glutathione (GSH). GSH is the main non-enzymatic antioxidant scavenger [54,55] and alterations in its synthesis, or in its pool, have been associated with neuronal cell death [56]. Fibroblasts derived from the healthy volunteer showed significantly higher total intracellular GSH levels compared to fibroblasts carrying the SOD1 mutations (Figure 4B). These findings suggest that affected individuals may lack GSH protective activity [24].

### 3.4. Bioenergetic Alterations in SOD1^L145F^ and SOD1^S135N^ Fibroblasts

#### 3.4.1. Cell Energy Phenotype Reveals a Different Response to Stress in Cells Expressing Mutated SOD1

Metabolic dysfunctions play an important role in ALS pathogenesis. Since SOD1 mutations affect mitochondrial functionality, we assessed the ability of cells to cope with the energy demand upon stress conditions. We added oligomycin, an ATP synthase inhibitor, together with the mitochondrial uncoupling agent FCCP. Analyses were conducted using Agilent Seahorse.

**Figure 5:**
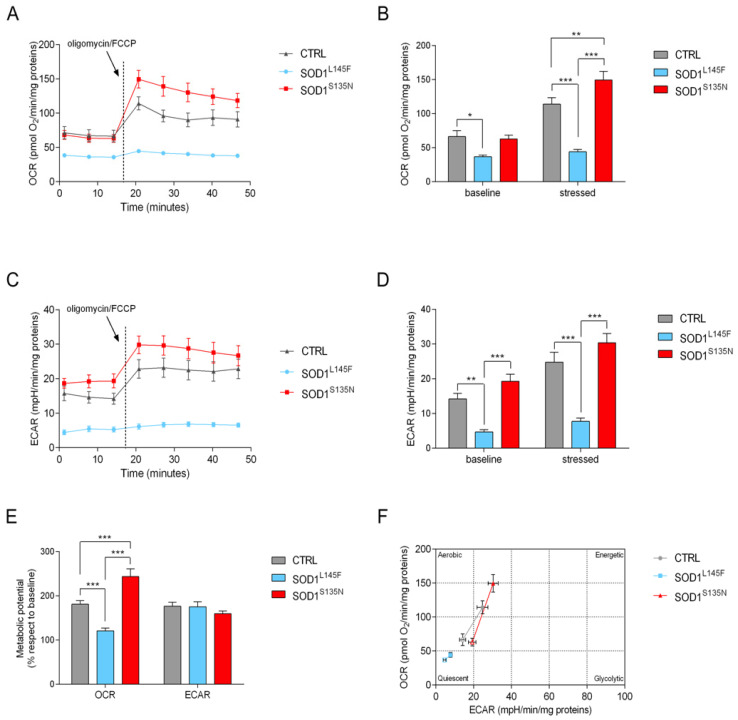
Cell energy phenotype evaluation. Representative OCR (**A**) and ECAR (**C**) profiles, expressed as pmol O_2_/min/mg proteins and mpH/ min/mg proteins in the control and SOD1 mutant cells, respectively. The arrows indicate the time of the simultaneous addition of oligomycin/FCCP. Baseline and stressed level of mitochondrial respiration (**B**) glycolysis (**D**) and metabolic potential. (**E**) Energetic panel of control and SOD1 mutants; open symbols represent baseline conditions and closed symbols the stressed ones (**F**). Bars indicate the mean ± SEM of at least three biological replicates. Statistical significance: * *p* < 0.05, ** *p* < 0.01 and *** *p* < 0.001.

The results are shown in Figure 5. No differences were observed in the basal OCR between SOD1^S135N^ and the control fibroblasts whose OCR and ECAR were already higher compared to the SOD1^L145F^ fibroblasts. However, under stress conditions we observed a strong increase in the parameters of both SOD1^S135N^ and the control fibroblasts, while the SOD1^L145F^ cells did not show any significant variation (Figure 5A–F). Our results suggest that, under stress conditions, the mitochondrial respiration rate was not increased in fibroblasts carrying the SOD1L145F, as was expected in order to meet the high energy demand. By subtracting the baseline value to the stressed one, we evaluated the cellular metabolic potential, i.e., the ability of the cells to meet the energy demand via respiration and glycolysis. On the one hand, the metabolic potential related to OCR (mitochondrial respiration) was significantly higher in the control and the SOD1^S135N^ fibroblasts than in SOD1^L145F^ cells, thus indicating that these cells lacked the ability to upregulate mitochondrial respiration under stress conditions (Figure 5E). On the other hand, SOD1^S135N^ fibroblasts showed increased OCR metabolic potential compared to the control fibroblasts. No significant differences between control and mutant fibroblasts were observed for ECAR, thus suggesting that the ability to upregulate glycolysis was quite similar. Our results, taken together, show that SOD1^L145F^ cells have a more quiescent phenotype than both SOD1^S135N^ and the control fibroblasts (Figure 5F).

#### 3.4.2. Mito Stress Test Shows That SOD1^L145F^ Fibroblasts Work at Their Maximum Respiratory Rate

A major goal of current bioenergetic research is to study cellular and molecular processes in models of physiological relevance, while avoiding many of the artifacts associated with mitochondrial isolation and cell permeabilization. Our experimental setting, using intact cells as a model, allowed a more accurate representation of these physiological processes [57].

Metabolic assays were carried out to compare mitochondrial bioenergetics between SOD1 mutants and control fibroblasts through Agilent Seahorse XF Analyzers.

**Figure 6:**
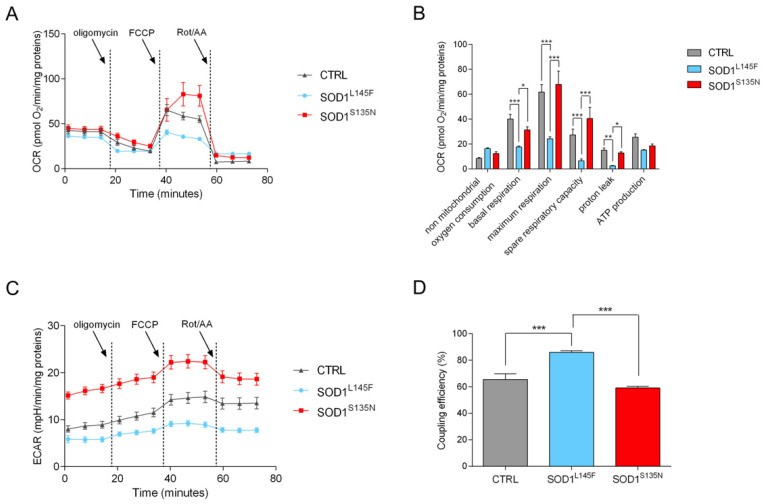
Mitochondrial functionality. OCR (**A**) and ECAR (**C**) traces, expressed as pmol O_2_/min/mg proteins and mpH/min/mg proteins in control and SOD1 mutant cells, respectively. Arrows indicate the time of addition of oligomycin, FCCP and rotenone/antimycin A. Analysis of key mitochondrial parameters (**B**) and coupling efficiency (**D**). Bars indicate the mean ± SEM of at least three biological replicates. Statistical significance: * *p* < 0.05, ** *p* < 0.01, *** *p* < 0.001.

As shown in Figure 6A,B, both the control and SOD1^S135N^ fibroblasts had significantly higher basal respiration and maximum respiration rates compared to the SOD1^L145F^ fibroblasts. Since this was the maximum rate of respiration that the cell could achieve under a “physiological energy demand”, this suggests that the SOD1^L145F^ fibroblasts had already reached their highest respiratory rate and were not able to increase any further.

In fact, the spare respiratory capacity, obtained by subtracting basal respiration to the maximum respiration rate, was significantly lower in SOD1^L145F^ cells, a further sign that these cells were already operating close to their bioenergetic limit. Moreover, a decrease in maximum respiratory capacity is a strong indicator of potential mitochondrial dysfunction, and an increased proton leak [58] can also be a sign of mitochondrial damage.

However, SOD1^L145F^ fibroblasts showed a very low rate of proton leak compared to both the control and SOD1^S135N^ fibroblasts, which behaved quite similarly.

The ECAR profile (Figure 6C) revealed higher values for the SOD^S135N^ fibroblasts, thus suggesting that these cells had a higher glycolytic rate.

Finally, the rate of mitochondrial ATP production was measured upon oligomycin injection: no significant differences were observed between the control cells and the mutant fibroblasts. The coupling efficiency ratio (CE ratio) is a useful indicator of the metabolic activity of mitochondria, as it is sensitive to changes in mitochondrial bioenergetics, and it is an internally normalized ratio. Our data showed a higher CE ratio in SOD1^L145F^ fibroblasts compared to both control and SOD1^S135N^ fibroblasts. (Figure 6D).

#### 3.4.3. SOD^S135N^ Fibroblasts Rely More on Glycolysis Than on Oxidative Phosphorylation for ATP Production

**Figure 7:**
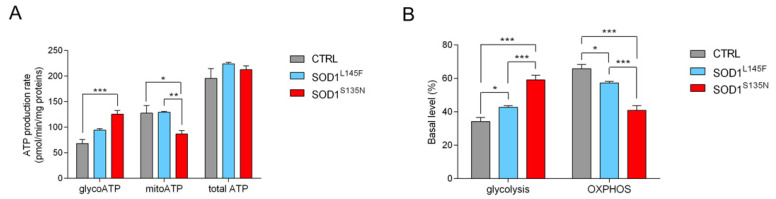
Evaluation of ATP production in SOD1 mutant fibroblasts. (**A**) ATP production rate expressed as pmol/min/mg protein and basal percentage level of glycolysis and oxidative phosphorylation (**B**). Data are shown as the mean ± SEM of at least three independent experiments. Statistical significance: * *p* < 0.05, ** *p* < 0.01, *** *p* <0.001.

The ATP levels were measured to assess the relative contributions of glycolysis and oxidative phosphorylation to the overall ATP production; the ATP synthase inhibitors oligomycin and Rot/AA, and inhibitor of mitochondrial complexes I and III were added in sequence. As reported in Figure 7A, no significant differences were detected in the overall levels of ATP between the cells carrying mutated SOD1 and the control fibroblasts. Both of the SOD1 mutations determined a higher basal glycolytic level than the control cells; however, while both SOD1^L145F^ and control fibroblasts still produced most of their ATP through oxidative phosphorylation, only SOD1^S135N^ fibroblasts produced the majority of their ATP through glycolysis, showing a shift towards the Warburg effect (Figure 7B).

#### 3.4.4. SOD1^S135N^ Fibroblasts Show Higher Glycolytic Rate Than the Control

Given the results obtained in the analysis of the cell energy phenotype, as well as the ECAR profile of the mito stress test, we hypothesized glycolysis might be increased in the SOD1^S135N^ cells. We assessed glycolytic functions, glycolytic rate, and compensatory glycolysis using the Agilent Seahorse XF Glycolysis Stress Test Kit and the Agilent Seahorse XF Glycolytic Rate Assay Kit.

**Figure 8:**
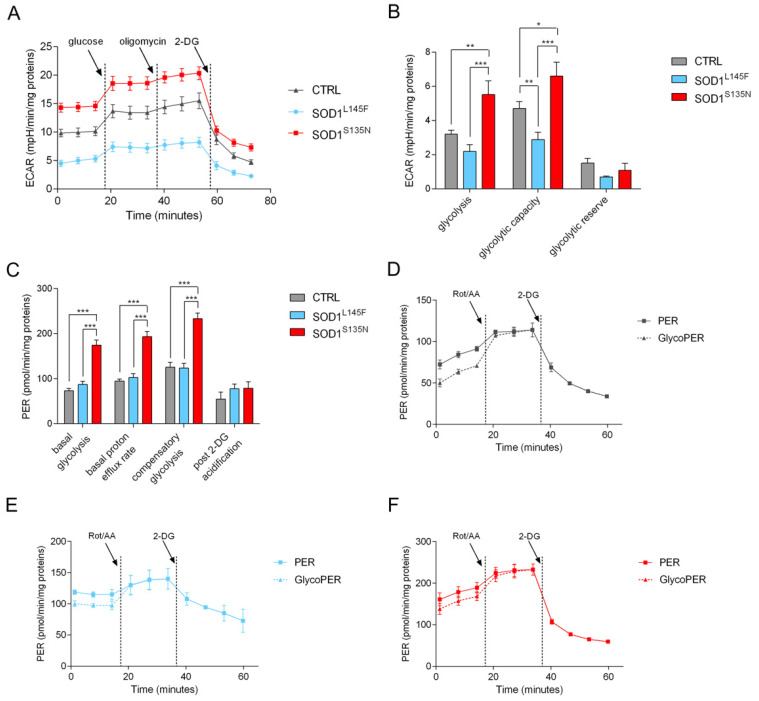
Glycolysis evaluation. (**A**) Representative ECAR profiles of control and SOD1 mutant cells of at least three independent experiments, expressed as mpH/min/mg proteins. The arrows indicate the time of addition of glucose, oligomycin and 2-DG. (**B**) Analysis of different glycolytic parameters. (**C**) Analysis of basal and compensatory glycolytic levels in control and SOD1 mutant cells. Representative PER and glycoPER profiles of control cells (**D**), SOD1^L145F^ (**E**), and SOD1^S135N^ (**F**) mutant cells expressed as pmol/min/mg protein. The arrows indicate the time of rotenone/antimycin A and 2-DG addition. Results are expressed as the mean ± SEM of at least three independent experiments. Statistical significance: * *p* < 0.05, ** *p* < 0.01, *** *p* < 0.0013.

The ECAR profile, reported in Figure 8A, showed a higher proton extrusion both at the basal level and after the injection of a saturating glucose concentration into the SOD1^S135N^ fibroblasts, compared to both the control and SOD1^L145F^ fibroblasts. Subsequently, we modulated ECAR via the administration of oligomycin and hexokinase inhibitor 2-deoxyglucose (2-DG), and we assessed the glycolytic parameters. In particular, SOD1^S135N^ cells showed a significant increase in glycolysis, as well as in glycolytic capacity, compared to both the control and SOD1^L145F^ fibroblasts; on the other hand, SOD1^L145F^ cells showed the lowest values for all parameters (Figure 8B). Since cells, in response to environmental changes, can switch from glycolysis to oxidative phosphorylation for energy production, we measured both basal glycolytic rate and compensatory glycolysis following mitochondrial inhibition. SOD1^S135N^ fibroblasts showed higher basal glycolysis and proton efflux rate compared to the control and SOD1^L145F^ cells (Figure 8C–F). The addition of rotenone and antimycin A triggered a higher compensatory glycolysis in SOD1^S135N^ cells compared to the control, probably due to their higher glycolytic capacity and reserve. When 2-DG was added the PER was minimized, and the rate of acidification measured was found to be similar for all fibroblasts (Figure 8C).

## 4. Discussion

ALS is characterized by clinicopathological variability. This inherent feature of the disease hinders the identification of pathogenic mechanisms and hampers the validation of therapeutic strategies. The use of cells derived from fALS patients may help overcome these issues and become instrumental to identify impaired cellular functions and their possible correlation with ALS symptoms, genetic alterations, and disease stage. The use of induced pluripotent stem cells (iPSCs) enables the development of neural cells to model ALS; however, the technology to reprogram iPSCs and the protocols to induce their differentiation are still money- and time-consuming. Fibroblasts can be easily derived from ALS patients. Interestingly, several studies have shown that skin cells share a few typical ALS pathogenic features with neural cells, including the accumulation of proteins such as TDP-43 [59,60], FUS [61] and VCP [62], neuroinflammatory markers such as TNF-a [63] and IL-6 [64], and show increased levels of matrix metalloproteinases 2 and 9 [65].

Skin cells can be collected at different stages of the disease. This enables the assessment of the progression of the cellular impairments, along with the clinical course, as has been shown in animal models [66]. Consequently, fibroblasts represent an adequate study model to screen for and detect ALS-induced changes, and can then be further characterized in iPSC-derived neural cells.

In our study, we characterized the bioenergetic profile of fibroblasts derived from two fALS patients carrying mutations in SOD1, identified as SOD1^S135N^ [16] and SOD1^L145F^ [19]. The two mutations were associated with two symptomatically different forms of ALS. In both mutated fibroblasts cell lines, metabolic rearrangements were detected; however, our data suggest that different metabolic components might have been involved. Additional studies including multiple cell lines and isogenic controls are required to establish whether there is a causal link between observed metabolic changes and clinical manifestations in patients.

Our preliminary morphological and functional characterization has shown that cells derived from the two patients had a larger shape compared to the control fibroblasts, which typically showed a spindle-shape morphology. Interestingly, we observed that, in a few cells derived from ALS patients, the SOD1 protein was mostly detected in the cytoplasmic region around the nucleus. It has been recently suggested that the nuclear fraction of SOD1 may have a functional role as a defense mechanism against oxidative stress. In fact, SOD1 might act as a transcription factor in response to an increase in ROS levels, and induce the expression of oxidative resistance and repair genes [11]. In light of this, similarly to the fibroblasts of the SOD^G93A^ rat model [66], the accumulation of SOD1 in the perinuclear region might be explained by the inability of the mutated SOD1 to enter it. The role of SOD1 as transcription factor is currently being investigated; indeed, it might play a role in counteracting the effect of ROS at the cellular level. However, additional studies are required to confirm our observations concerning SOD1 distribution in patients’ fibroblasts and to understand whether there is correlation with the pathogenesis of ALS, or whether this simply reflects a different cell morphology.

Remarkably, ALS fibroblasts showed an increased proliferation rate. This increased proliferation rate, together with the activation of the ERK pathway, has recently been reported in fibroblasts and motor neurons bearing other mutations in SOD1 [52,66] and TDP43 [60]. However, it has not yet been established whether increased proliferation is triggered by a specific ALS-mutated protein, or whether it is a common trait to all disease-related mutations. Oxidative stress is a common ALS hallmark, and it might trigger cell proliferation through the ERK pathway [67,68]. In addition, Bains et al. hypothesized that oxidative stress-mediated neuronal loss might be initiated by a decline in the concentration of the antioxidant molecule GSH [23,24,69,70,71]. Consistently, both SOD1^S135N^ and SOD1^L145F^ fibroblasts showed very low levels of total glutathione compared to healthy control fibroblasts. This observation was matched by an increase in ROS level, although not statistically significant, which might be linked to the loss of function caused by both SOD1 mutations. Our results are consistent with previous studies which show imbalanced GSH homeostasis in several models of ALS [72]. Interestingly, lack of GSH or, more generally, of cysteines redox balance, could play a role in the pathogenesis of neurodegenerative diseases [73].

However, the metabolic profiles of the two mutants were different. Indeed, according to the Cell Energy phenotype test, SOD1^S135N^ cells showed an overall higher metabolic potential, compared to the control and SOD1^L145F^ cells. In fact, the SOD1^S135N^ fibroblasts increased their OCR and ECAR values in response to the stress conditions and showed a higher glycolytic rate at baseline (baseline ECAR) compared to the control and SOD1^L145F^ cells.

Mitostress experiments also confirmed the high basal ECAR, but showed that SOD1^S135N^ cells can only slightly increase their ECAR value upon FCCP addition. These data suggest that glycolysis does not compensate for mitochondrial stress, and it is already working at maximum rate. The ATP rate assay confirmed this hypothesis, since SOD1^S135N^ cells relied more on glycolysis than on oxidative phosphorylation for ATP production. Our data suggest a metabolic shift of the cells towards the Warburg effect. Glycolysis upregulation was strongly confirmed by all parameters measured in the Glycolitic rate assay. In the glycolitic stress test, when glucose was added, SOD1^S135N^ cell glycolysis did not significantly increase, nor did it for FCCP addition. On the whole, glycolysis appears to work at its maximum rate in the basal state, and is not to be able to compensate for mitochondrial stress.

SOD1^L145F^ fibroblasts showed a quiescent cell energy phenotype, being almost unable to meet energy demand via glycolysis when mitochondria were uncoupled, as demonstrated by the fact that ECAR did not increase with the addition of stressors. This was confirmed by the Mitostress assay profiles, showing a lower basal respiration rate compared to both the SOD1^S135N^ and the control cells, as well as a lower maximum respiration and spare respiratory capacity. This suggests dysfunctional glycolysis, depriving the cells of reduced substrates. On the other hand, mitochondria appeared fully coupled; in fact, the ATP rate assay showed that most ATP was produced by oxidative phosphorylation. Despite the presence of functional mitochondria, the SOD1^L145F^ fibroblasts could not efficiently use glucose; this might also explain their slightly lower proliferation rate with respect to SOD1^S135N^.

Several studies have shown that mutated SOD1 proteins might have an impact on mitochondrial functionality at several levels, including impaired ATP synthesis, depressed respiration rate, and increased cellular ROS production [18,74,75,76]. The mitochondrial impairments observed in SOD1^S135N^ cells were consistent with this line of evidence. Indeed, SOD1^S135N^ cells experiencing metabolic reframing might be caused by increased oxidative stress caused by the loss of SOD1 activity. The metabolic rearrangement observed in the SOD1^L145F^ cells suggests the prominent effect of the glycolysis mutation that appeared strongly downregulated. Our hypothesis, supported by our bioinformatics analyses, is that SOD1^L145F^ mutation might affect protein dimerization and lead to the accumulation of aggregates in the cytosol, impacting glycolysis.

Increased ROS has been demonstrated to lead to PGC-1a downregulation via the downregulation of the mitochondrial sirtuin SIRT3 [77]. Moreover, the downregulation of PGC-1a, a key regulator of mitochondria biogenesis, has already been reported in ALS patients, while PGC-1a overexpression has been shown to expand lifetime in G93A mice [78]. In fact, Buck and colleagues found SIRT3 to be downregulated in G93A mice. In turn, SIRT3 downregulation activates AMPK leading to mTOR inactivation [79]. Further studies will be necessary to assess whether the metabolic alterations of the SOD1^L145F^ fibroblasts imply AMPK activation and mTOR inhibition to a higher extent than the SOD1^S135N^ mutant, which compensates for mitochondrial dysfunction by upregulating glycolysis. Common features of metabolic dysregulation may be shared by many neurodegenerative diseases that show mitochondrial impairment. Previous work by Sonntag and coworkers [80] has shown that fibroblasts derived from patients affected by late-onset Alzheimer’s disease have an impairment in mitochondrial potential caused by a decrease in NAD metabolites, which leads to an increase in glycolysis. Moreover, Aldana and collaborators [81] found a dysregulation of glutamate–glutamine homeostasis in iPSC-derived neurons from frontotemporal dementia patients.

## 5. Conclusions

In conclusion, our study is a multilayered analysis of the impairments determined by two non-classical mutations (p.S135N and p.L145F) in the SOD1 protein. We showed that each mutation affects the protein structure differently, matching its impact on the morphological, functional and energetic properties of patient-derived fibroblasts. Of note, the mutation with the most severe phenotypes at the protein and cellular level also corresponded to the most aggressive form of ALS. In order to corroborate the results and establish a proper link between the unbalancing of the energy metabolism, the clinical phenotype, and prognosis, further investigations—including a larger cohort of control and ALS-patient-derived fibroblast lines—are needed.

## Data Availability

Data is contained within the article.
